# Supplementary material for: Amyloid Aggregates of Smooth-Muscle Titin Impair Cell Adhesion
Source: Int J Mol Sci. 2021 Apr 27;22(9):4579. doi: 10.3390/ijms22094579 (PMC8123791; doi:10.3390/ijms22094579)
Supplement: Supplementary file 1 [file ijms-22-04579-s001.zip › ijms-1186479-supplementary.pdf]

Supplementing Materials

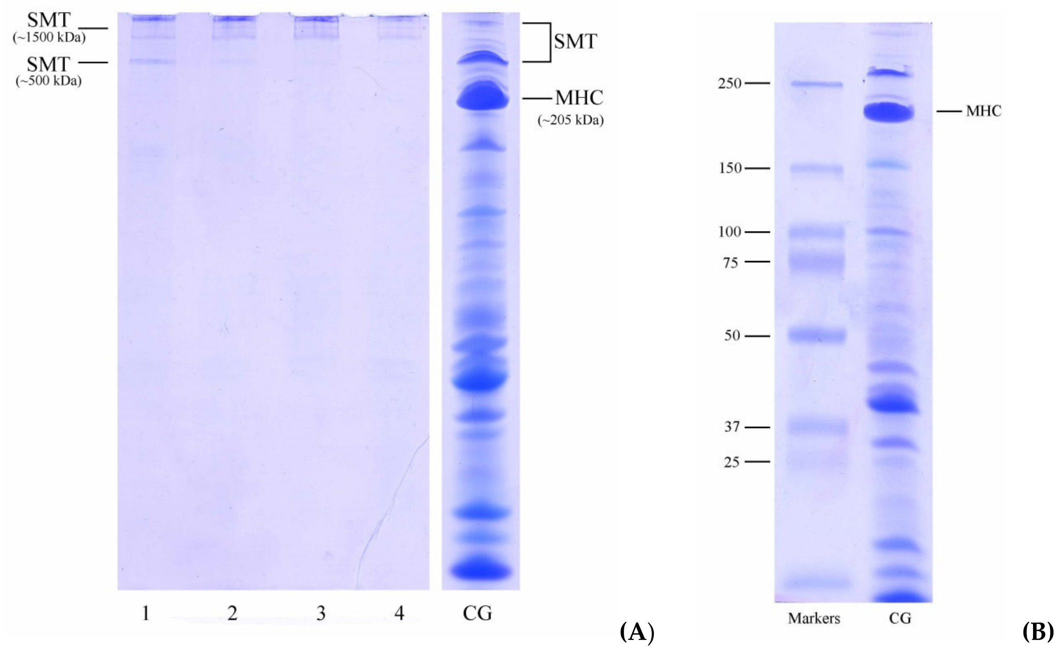

Supplementary Fig. S1. **(A)** SDS-PAGE of purified SMT (lanes 1–4). Chicken gizzard was used as a control (right-hand lane). **(B)** Protein markers of molecular weights within the range of 10–250 kDa were also used (left-hand lane). MHC, myosin heavy chains; CG, chicken gizzard, SMT, smooth muscle titin.

Supplementary Table S1. The list of detected titin peptides

Sequence	Mass	Proteins	Start position	Gene names	Unique (Proteins)	Charges	Score	Intensity	MS/MS Count
EPGHLEESYAQQTTLEYGYK	2342.0703	Q8WZ42	719	TTN	yes	3	96.447	5562000	1
IDSTVSQDSAWYTATAINK	2069.9906	Q8WZ42	1617	TTN	yes	2;3	69.602	5750400	2
AINIAGETSSHAFLLVQAK	1969.0633	Q8WZ42	2153	TTN	yes	3	79.013	12857000	3
EPATITEEAVSIDVTQGDPATLQVK	2611.3229	Q8WZ42	5504	TTN	yes	3	53.266	10740000	2
ADNSVGAVASSAVLVIK	1599.8832	Q8WZ42	8021	TTN	yes	2	62.891	2878200	1
PVSVDLALGESGTFK	1518.793	Q8WZ42	8145	TTN	yes	2	133.05	4020000	1
MTLVENTATLTVLK	1532.8484	Q8WZ42	8187	TTN	yes	2	116.55	2474100	1
DNDAVFEIDIK	1277.6139	Q8WZ42	9597	TTN	yes	2	85.355	3024200	1
MFFASHTEEEVSVTVPEVQK	2293.0936	Q8WZ42	10174	TTN	yes	3	61.102	3315400	1
LDQAGEVLYQALNAITTAILTVK	2444.3526	Q8WZ42	12387	TTN	yes	3	74.876	44910000	3
HILVINDSQFDDEGVYTAEVEGK	2577.2235	Q8WZ42	12466	TTN	yes	3	64.295	2515200	1
ESAVFTVELSHDNIR	1715.8479	Q8WZ42	12871	TTN	yes	3	85.457	3173700	1
NLANIEVSETDTIK	1545.7886	Q8WZ42	13395	TTN	yes	2	150.46	4860000	1
TISGEIDVNVIAR	1385.7514	Q8WZ42	14402	TTN	yes	2	127.76	9556300	2
VTDVIEGTEVQFQVR	1718.8839	Q8WZ42	15172	TTN	yes	2	147.91	5780100	2
VSAVNAAGEGPPGETQPVTVAEPQEPPAVELDVSVK	3567.7944	Q8WZ42	16003	TTN	yes	3	23.059	2946300	1
GYIVEMQEEGTTDWK	1784.7927	Q8WZ42	16665	TTN	yes	2	84.479	3066200	1
EFMEVEEGTNVNIVAK	1807.8662	Q8WZ42	17051	TTN	yes	2	184.24	3545600	1
TDEHYTVETDNFSSVLTIK	2198.0379	Q8WZ42	18480	TTN	yes	3	97.284	4356400	1
NIVGEVGDVITIQVHDIPGPPTGPIK	2664.4487	Q8WZ42	22069	TTN	yes	3	72.881	0	1
VTGLTEGNEYEFHVMAENAAGVGPASGISR	3062.4404	Q8WZ42	29141	TTN	yes	3	48.422	45431000	3
AMIATSETHTELVIK	1642.86	Q8WZ42	31108	TTN	yes	3	56.482	4227900	1
FGVTITVHPEPHVTWYK	2010.0363	Q8WZ42	32636	TTN	yes	3	99.451	10930000	2
STFEISSVQASDEGNYSVVVENSEGK	2761.2566	Q8WZ42	33543	TTN	yes	3	136.4	73879000	6

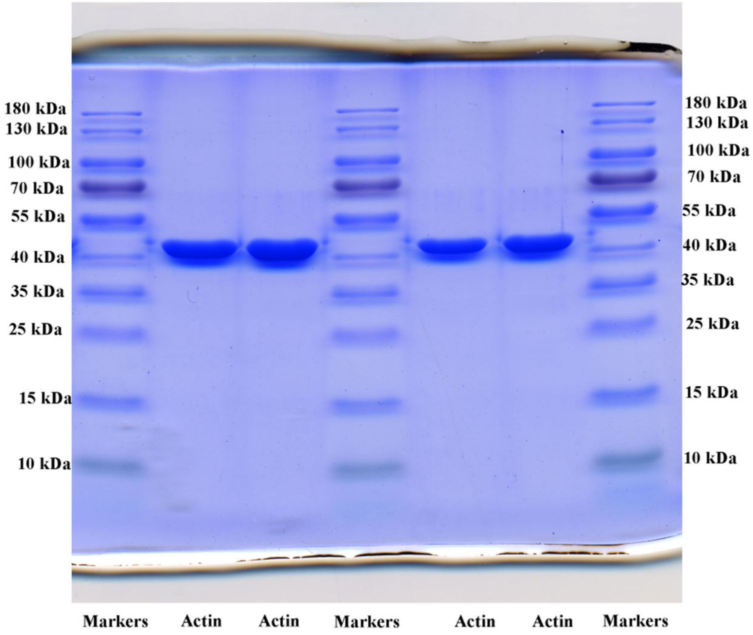

Supplementary Fig. S2. SDS-PAGE of purified actin and the protein markers of molecular weights within the range of 10–180 kDa were also used.
